# Gas-Phase Synthesis of Iron Silicide Nanostructures Using a Single-Source Precursor: Comparing Direct-Write Processing and Thermal Conversion

**DOI:** 10.1021/acs.jpcc.3c08250

**Published:** 2024-02-08

**Authors:** Felix Jungwirth, Alba Salvador-Porroche, Fabrizio Porrati, Nicolas P. Jochmann, Daniel Knez, Michael Huth, Isabel Gracia, Carles Cané, Pilar Cea, José María De Teresa, Sven Barth

**Affiliations:** † Institute of Physics, Goethe University Frankfurt, Max-von-Laue-Str. 1, Frankfurt am Main 60323, Germany; ‡ Institute for Inorganic and Analytical Chemistry, Goethe University Frankfurt, Max-von-Laue-Str. 7, Frankfurt 60438, Germany; § Instituto de Nanociencia y Materiales de Aragón (INMA), CSIC−Universidad de Zaragoza, Zaragoza 50009, Spain; ∥ Institute of Electron Microscopy and Nanoanalysis, Graz University of Technology, Steyrergasse 17, Graz 8010, Austria; ⊥ Institut de Microelectrònica de Barcelona (IMB), Centre Nacional de Microelectrònica (CNM), Consejo Superior de Investigaciones Científicas (CSIC), Barcelona 08193, Spain; # Laboratorio de Microscopías Avanzadas (LMA), Universidad de Zaragoza, Edificio de I+D+i, Campus Río Ebro, Zaragoza 50018, Spain

## Abstract

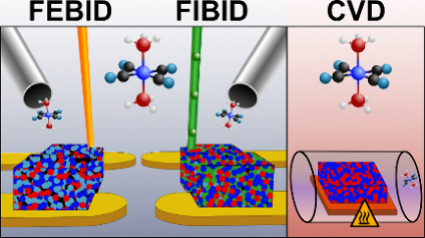

The investigation of precursor classes for the fabrication of nanostructures is of specific interest for maskless fabrication and direct nanoprinting. In this study, the differences in material composition depending on the employed process are illustrated for focused-ion-beam- and focused-electron-beam-induced deposition (FIBID/FEBID) and compared to the thermal decomposition in chemical vapor deposition (CVD). This article reports on specific differences in the deposit composition and microstructure when the (H_3_Si)_2_Fe(CO)_4_ precursor is converted into an inorganic material. Maximum metal/metalloid contents of up to 90 at. % are obtained in FIBID deposits and higher than 90 at. % in CVD films, while FEBID with the same precursor provides material containing less than 45 at. % total metal/metalloid content. Moreover, the Fe:Si ratio is retained well in FEBID and CVD processes, but FIBID using Ga^+^ ions liberates more than 50% of the initial Si provided by the precursor. This suggests that precursors for FIBID processes targeting binary materials should include multiple bonding such as bridging positions for nonmetals. In addition, an in situ method for investigations of supporting thermal effects of precursor fragmentation during the direct-writing processes is presented, and the applicability of the precursor for nanoscale 3D FEBID writing is demonstrated.

## Introduction

State-of-the-art synthesis and integration of nanomaterials is often based on top-down approaches to build nanodevices in high resolution.^1^ Among these processes, popular approaches to creating 2D nanostructures and patterns with a very high resolution are based on focused beams of charged particles such as electron-beam lithography (EBL)^2^ or focused-ion-beam milling.^3^

In addition, the maskless site-selective writing of nanostructures with the desired shape and dimension using focused electron/ion beams is a powerful tool for bottom-up nanofabrication.^4−6^ While a large variety of approaches based on additive manufacturing have reached a high level of sophistication for objects down to the lower micrometer range, challenges remain for the preparation of 3D nanostructures.^7,8^ The general trend of miniaturization of devices and functional 1D–3D structures requires continuous progress enabling the development of novel applications due to specific functionalities emerging at the nanoscale (e.g., plasmonics, magnetic phenomena).^4,9−19^ Therefore, both techniques, focused- electron-beam- and focused-ion-beam-induced deposition (FEBID/FIBID), are of particular interest. General introductions to the subject, including the physics of beam–substrate interactions^6,20,21^ and suitable precursors for FEBID/FIBID,^22−24^ are available. The main differences between the exclusively additive FEBID and the more complex FIBID are the incorporation of ions into the growing material, implantation into the substrate, and damage to the substrate material either by amorphization or localized sputtering of the substrate/deposit due to the momentum transfer of the ions.^25,26^ For example, Ga^+^ ion sources inherently result in the incorporation of Ga into the growing material and thus in a material composition that is dependent on the growth rate.^27−29^ Inadvertent incorporation of the ion source material into the deposit can be prevented by using alternatives such as gas field ion source processing for FIBID.^30,31^ While similar effects occur for different ions, the specific contributions to sputtering, energy transfer, and fragmentation efficiency of precursor moieties is determined by ion mass, size, and energy.^25,32^

Latest results of electron-induced fragmentation from surface science studies, with relevance regarding the fundamentals of the FEBID process, suggest a partial fragmentation of precursors in the first step. In the case of metal carbonyls, this is followed/accompanied by thermal fragmentation and CO release or electron-induced CO cleavage leading to composites.^22^ It should be noted that the highest metal contents obtained in FEBID coincide with surface science studies showing low-temperature thermal fragmentation of the formed intermediates. Thermal effects on the deposits’ composition have been demonstrated in surface science studies on partially electron-fragmented M_*x*_(CO)_*y*_ films formed by electron-irradiated Ni(CO)_4_,^33^ Fe(CO)_5_,^34^ and HFeCo_3_(CO)_12_^35^ layers and annealing at substrate temperatures below 60 °C.

Moreover, similar surface science studies have demonstrated that ion bombardment of condensed precursor layers results in films with a much higher metal content, when compared to pure electron irradiation.^36,37^ In addition, the studies do not describe a preferential sputtering of either metal in bimetallic CpFe(CO)_2_Re(CO)_5_ by Ar^+^ ion bombardment of condensed precursor films but rather C_5_FeRu layer formation with carbonyls being liberated efficiently.^37^ This is in line with FIBID of a 2:1 Co:Si metal/metalloid ratio from H_2_Si(Co(CO)_4_)_2_.^38^ In contrast, H_3_SiCo(CO)_4_-derived FIBID material revealed significant Si loss, while FEBID material retained the metal/metalloid ratio, which was attributed to increasing impact of sputtering effects in FIBID due to low growth rates.^38,39^

In the past, FIBID fragmentation was attributed to a thermal spike model or a binary collision model.^26^ However, the actual process is apparently much more complicated. A comprehensive description of FIBID should also include the differences in the ions used for the deposition and should consider the contributing factors of secondary electron-induced deposition.

The deposit composition can also be altered by the number of electrons/ions relative to the precursor concentration during the deposition process, which determines a specific deposition regime in which actual FEBID/FIBID growth takes place. In FEBID, ineffective precursor fragmentation can be caused by (i) ligand incorporation due to insufficient cleavage and a very high growth rate for electron-limited decomposition or (ii) unintended ligand fragmentation leading to more byproducts in the precursor-limited regime.^6^ Typically, conditions in between the limiting regimes will produce the lowest levels of contaminants/ligand fragments in FEBID materials.^40^ As mentioned before, the FIBID process is more complex and can be viewed as a balance between material deposition through precursor fragmentation and sputtering/milling of surface atoms. In the ion-limited regime, the precursor molecules are decomposed primarily by momentum or local heat transfer, which is accompanied by electron-induced fragmentation by secondary electrons generated by the ion impact on the substrate.^41^ In the precursor-limited regime, the molecular sources are fragmented, resulting in a deposit, but at the same time, sputtering will remove parts of the deposit or substrate material.^41^ Differences in the ion beam scanning strategies will impact the final appearance of the deposit morphology such as the formation of tubular nanostructures in FIBID instead of a solid nanowire as observed for FEBID, when single-spot deposition is used.^42−47^

The study presented here targets the formation of metal silicides, which are intermetallic compounds of metals and silicon. Metal silicides form a significant, structurally complex, and compositionally adaptable group of inorganic solids with a broad range of electronic, magnetic, optical, catalytic, and mechanical properties.^48−50^ More specifically, besides their well-established use as deoxidizers in steel manufacturing, iron silicides have great potential as materials for optoelectronics, electronic circuits, spintronics, and data storage and even as battery components.^51−54^

Herein, we compare three gas-phase processes for the conversion of a single-source precursor, (H_3_Si)_2_Fe(CO)_4_ with an Fe:Si ratio of 1:2. This comparison enables the examination of the potential impact of thermal fragmentation in the conversion of the molecule during direct-write processes. The influence of precursor pressure, ion/electron flux (current), and acceleration voltage on the composition of the deposits is evaluated. The physical characteristics and microstructural features of the deposits differ significantly with the molecule-to-material conversion technique employed. The findings offer insights into the influence of metal to silane ligand bonding on fragmentation during ion- and electron-induced deposition. The observed composition of materials from electron-induced fragmentation is supplemented with low-pressure thermal CVD results as a benchmark for exclusively thermal decomposition. Besides the in-plane deposition, 3D writing of nanowires has been demonstrated by FEBID. Finally, we suggest a micromembrane-based approach that allows for the investigation of thermolabile intermediates in FEBID by quick in situ pulsed cycling of deposits below the thermal decomposition temperature.

## Methods

### Precursor Synthesis

Sodium, benzophenone, Fe(CO)_5_, H_3_Si(C_6_H_5_), iodine, pentane, hexane, and diethyl ether were purchased from Sigma-Aldrich. 1,2,3,4-Tetrahydronaphthalene was purchased from ABCR. Pentane, hexane, diethyl ether, and 1,2,3,4-tetrahydronaphthalene were dried over sodium and degassed by three freeze–pump–thaw-cycles. Na_2_[Fe(CO)_4_] was prepared in diethyl ether by titration of a sodium/benzophenone mixture and Fe(CO)_5_. The insoluble Na_2_[Fe(CO)_4_] was filtered, washed twice with hexane, and dried under dynamic vacuum at room temperature. Anhydrous H_3_SiI was synthesized by reaction of H_3_Si(C_6_H_5_) at temperatures of 233–238 K using pure HI, which was in situ prepared by reaction of iodine with 1,2,3,4-tetrahydronaphthalene. Purification was carried out by triple distillation at atmospheric pressure under an inert gas using a Vigreux column. NMR spectra were recorded using a Bruker AV500 and a Bruker DPX 250 nuclear magnetic resonance (NMR) spectrometer at room temperature and were referenced to SiMe_4_ (TMS).

The synthesis of (H_3_Si)_2_Fe(CO)_4_ was carried out by salt elimination using H_3_SiI and Na_2_[Fe(CO)_4_] in pentane similar to a published procedure.^55^ Typically, 3.26 g (15.3 mmol) of dried Na_2_[Fe(CO)_4_] was dispersed in ∼20 mL of pentane at 233 K. Subsequently, 4.35 g (27.5 mmol) of H_3_SiI was added, and the mixture was allowed to warm up to ∼293 K. The solvent was removed under reduced pressure and at temperatures in the range 200–233 K. Evaporation temperatures of 233–298 K were used to collect the crude product in a cold trap maintained in liquid nitrogen. In order to ensure the complete removal of the solvent and iodosilane, the sublimation was repeated two times and the product was collected at a temperature range of 243–258 K of the crude product, yielding a colorless solid with a high vapor pressure. NMR was used for characterization. ^1^H NMR (500 MHz, C_6_D_6_, 298 K, TMS): 3.71 ppm (d; 3H; ^1^J(^1^H, ^29^Si) = 201 Hz; (*H*_*3*_Si)_2_Fe(CO)_4_); ^29^Si NMR (99 MHz, C_6_D_6_, 298 K, TMS): −57.0 ppm (q; 1Si; ^1^J(^1^H, ^29^Si) = 201 Hz; (H_3_*Si)*_*2*_Fe(CO)_4_).

### CVD Process

CVD was carried out in a homebuilt cold-wall reactor using high-frequency heating of a graphite susceptor for indirect heating of sapphire (0001) and Si (911) substrates. The substrates were attached to the graphite susceptor with silver paste to ensure efficient thermal contact. Substrate temperatures were limited to 573–773 K. The precursor was introduced into the reactor through a glass flange applying dynamic vacuum (∼10^–6^ mbar) while keeping the precursor temperatures in the range of 243–253 K. Typically, 30–40 mg of the precursor was used as a source for the growth experiments, and the growth was carried out for 30–60 min. A similar CVD setup has been described in the literature for the growth of thin films and nanostructures using molecular sources.^56,57^ In addition, micromembranes with integrated Joule heaters^58^ have been used inside an SEM microscope and the gas injection system provided the precursor as described below.

### FIBID Process and FEBID Sample Preparation

FIBID and FEBID were performed by using a dual-beam SEM microscope/focused ion beam (FIB) (FEI, Nova NanoLab 600) equipped with a Ga^+^ ion source. Generally, serpentine patterning strategies were adapted for the typical in-plane deposits described herein. The substrates used in the study are either (i) (0001)-oriented sapphire single crystals coated with a 250 nm Au film with an 8 nm Cr adhesion layer, (ii) (0001)-oriented sapphire single crystals coated with an ∼200 nm Cu film, or (iii) p-doped (100) Si with a 300 nm SiO_2_ top layer. Air-plasma cleaning was always performed in order to reduce the hydrocarbon level within the microscope’s chamber after the substrate was mounted. Prior to deposition experiments, the system was pumped for at least 48 h and the residual water content was reduced by using a Meissner trap for 4 h. This procedure allowed achieving a background pressure of <3.6 × 10^–7^ mbar. The precursor container was kept at 253 K for (H_3_Si)_2_Fe(CO)_4_ using an ethanol cooling bath to retain the vapor pressure at a suitable level. The total pressure within the deposition chamber during the process was regulated via a needle valve and typically kept at 1 × 10^–6^ mbar. The precursor was stored at 243 K and allowed to reach the temperature for deposition 2 h prior to the actual experiments.

FIBID process parameters, such as ion beam current (1–30 pA) and voltage (10–30 kV), were varied to study the effects on the deposits’ properties. The pitch (30 nm in the *x* and *y* directions) between deposition events and the dwell time (0.2 μs) were kept constant. The distance between the injection capillary and substrate was ∼100 μm, while the angle between the capillary and substrate was 35°.

The dimensions of deposits for two-point transport measurements were 5 μm × 1 μm with a height in the range of 20–140 nm for FIBID.

For the FEBID experiments, the capillary was positioned 100 μm laterally and vertically from the intended deposition spot on the substrate at a substrate–capillary angle of 15°. FEBID samples were prepared at acceleration voltages in the range of 5–20 kV, while varying the electron-beam current from 0.4 to 6.3 nA to study the effects on the deposits’ properties. For all samples, the pitch was set to 20 nm in the *x* and *y* directions and the dwell time was set to 1 μs.

Deposits for EDX were typically 1.4 μm × 1.4 μm and ∼200 to 300 nm high. The deposits for two-point transport measurements were 4.0–5.2 μm × 1 μm with a height in the range 90–100 nm.

### Deposits’ Chemical and Microstructural Characterization

The topographical features of the deposits were determined by atomic force microscopy (AFM) performed in noncontact dynamic force mode (Nanosurf, Easyscan2). The cantilever tip used had a radius of less than 7 nm (Nanosensors PPP-NCLR). The sample composition was characterized by energy-dispersive X-ray analysis (EDX) at a beam energy of 15 kV.

The thickness of the deposits used for the EDX investigations was large enough to avoid prominent contributions from the substrate layer. The error bars provided are the deviations between several EDX spectra recorded for deposits by using the predefined set of deposition parameters. In addition, the standardless quantification provides an estimate of the actual composition and will not be as accurate as EDX using defined material compositions for calibration. A slight overestimation of the carbon content could be caused by additional carbon deposition during EDX associated with residual carbon sources in the background gas. Additional spectra recorded on the bare substrate indicate no significant parasitic carbon deposition under the presented conditions here.

Lamellae for cross-sectional TEM analyses of the deposits were prepared via a standard FIB milling procedure utilizing Ga^+^ ions and MeCpPtMe_3_ as a precursor in an FEI HELIOS 650 FIB/SEM dual-beam microscope. The lift-out and initial milling step were carried out with an acceleration voltage of 30 kV, and the final thinning step was performed at 5 kV. The resulting lamellae were mounted onto an Omniprobe copper-based lift-out grid and transferred to the TEM microscope. TEM observations were carried out on an analytical Titan^3^ G2 60-300 instrument (FEI Company) operated at 300 kV in scanning (STEM) mode. The microscope was equipped with a windowless four-quadrant Super-X detector for EDX. High-angle annular dark-field (HAADF) imaging and EDX measurements were carried out along the nanostructure thickness. Data acquisition and analysis was performed using Gatan Microscopy Suite (version 3.6), and EDX data acquisition and analysis was performed with Velox by Thermo Scientific (versions 2.15 and 3.5) and AZtec (version 4.3) by Oxford Instruments, respectively. Carbon and oxygen were not included in the quantification due to the noticeable overall C deposition during TEM and the potential oxidation of the FeSi_*x*_ material during lamella storage and TEM investigation. Hence, the discussion is limited to qualitative discussion of the enrichment of the light elements within the deposits.

### Electrical Transport

Au microelectrodes for electrical characterization were prepared by standard ultraviolet contact photolithography and sputtering of an 8 nm Cr adhesion layer, followed by 250 nm Au for general substrates and 75 nm Au for the microelectrodes on SiO_2_ (300 nm)/p-Si substrates (CrysTec GmbH; Germany).

In situ two-point electrical transport measurements were carried out inside the SEM microscope after FEBID/FIBID.^59^ Standard measurements were performed using a Keithley 2400 source meter and an Agilent 34420A nanovoltmeter.

## Results and Discussion

**Figure 1:**
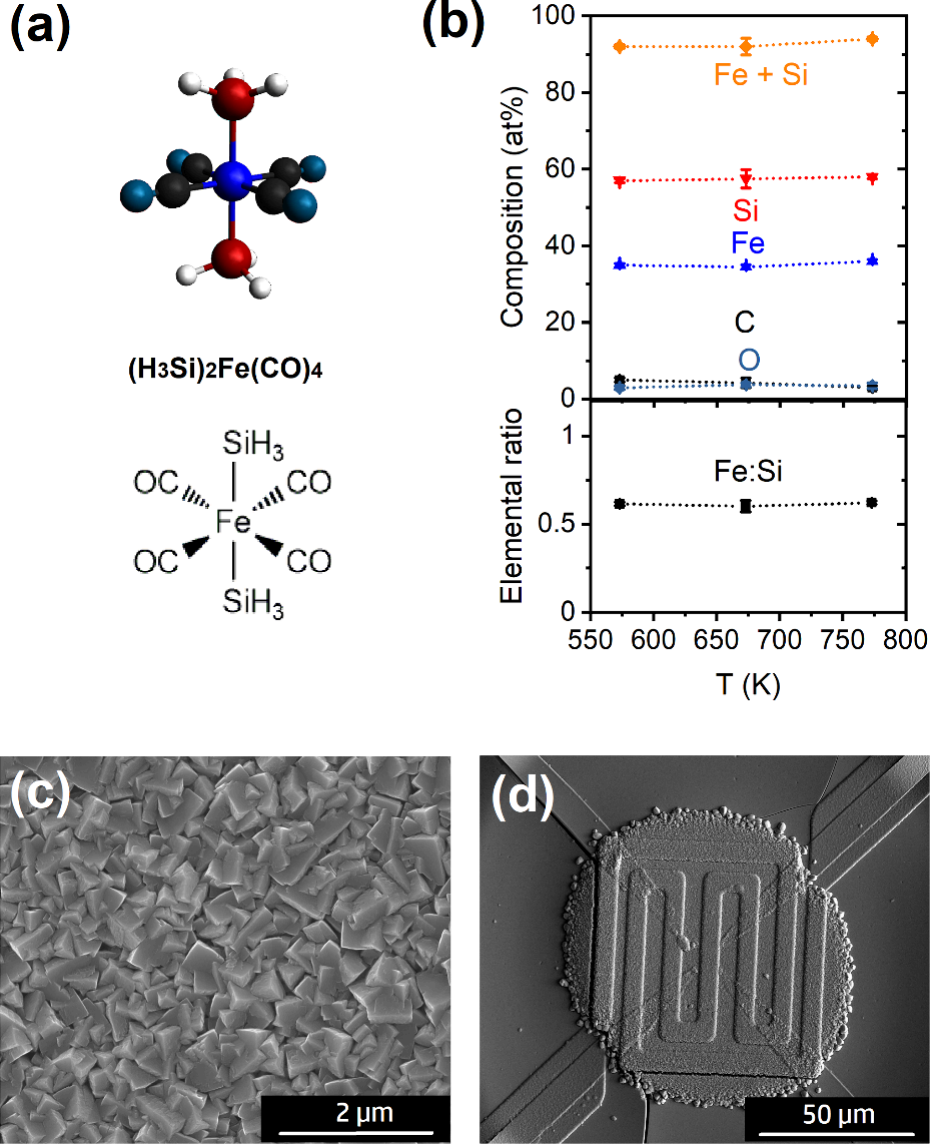
(a) Schematic illustrations of the (H_3_Si)_2_Fe(CO)_4_ single-source precursor used in this study. (b) Composition of CVD coatings on sapphire (0001) prepared at different temperatures using the (H_3_Si)_2_Fe(CO)_4_ precursor as determined by EDX. The elemental ratio of Fe:Si is also included. SEM images showing (c) a homogeneous FeSi-based CVD coating deposited at 773 K on sapphire (0001) in a homebuilt CVD cold-wall CVD reactor and (d) a deposit grown in an SEM chamber on a microhot plate at a similar temperature.

A schematic representation of the monomeric single-source precursor (H_3_Si)_2_Fe(CO)_4_ used in this study is shown in Figure 1a. The precursor contains four Fe–CO and two Fe–Si bonds, while the SiH_3_ moiety can be considered a ligand contributing to silicide formation.

The thermal decomposition of (H_3_Si)_2_Fe(CO)_4_ was investigated in a low-pressure cold-wall CVD reactor at substrate temperatures of 573–773 K and precursor temperatures of 243–253 K. The CVD growth resulted in thin films of a silver metallic appearance, which were deposited on Si (911), on sapphire (0001) single crystals, and on SiO_2_-based micromembranes. The composition was obtained from at least three different films prepared with identical parameters. According to the EDX data shown in Figure 1b, the deposits contain under all conditions a metal/metalloid content of more than 90 at. %, while a slight overestimation of C and O can be considered due to surface oxidation and absorption of hydrocarbons during storage and transfer to the SEM microscope for EDX characterization. The thermal decomposition via CVD retains 85% of the originally supplied Si in the deposit, which is equivalent to an Fe:Si ratio of 1:1.7 (0.6) in Figure 1b.

Figure 1c shows a typical SEM image of a film grown at 773 K with well-defined facets. As expected from the SEM image, a highly crystalline β-FeSi_2_ phase with a preferential growth direction (Figure S1 of the Supporting Information) is obtained. Similar results have been suggested in the literature, but no elemental composition has been provided.^60^ Deposits prepared at 573 K are predominantly amorphous but with a similar composition (Figure S1). It should be noted that the deposition on micromembranes containing integrated heating elements and Pt surface electrodes was also carried out by SEM, resulting in the same CVD coatings (Figure 1d). These platforms and their applicability in FEBID studies will be discussed in more detail; vide infra.

**Figure 2:**
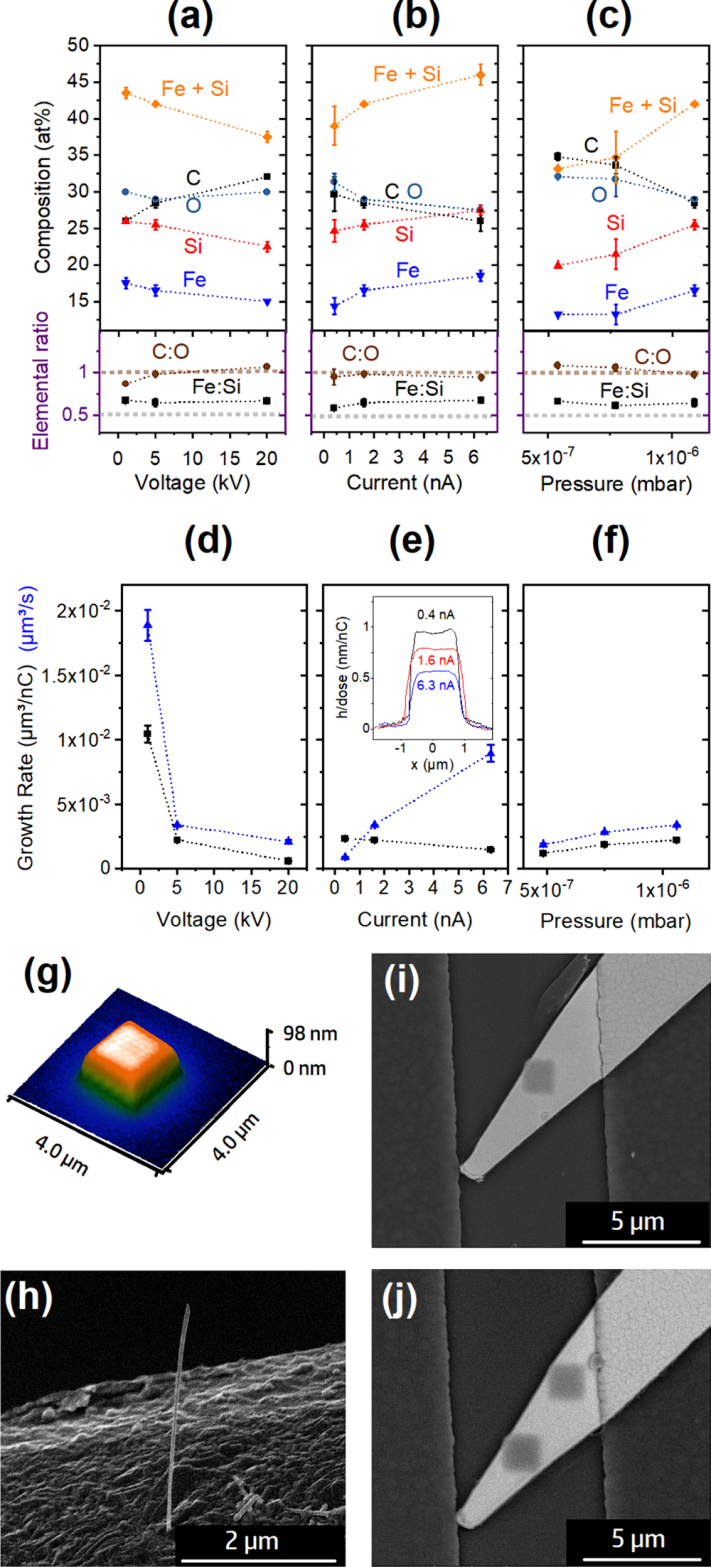
The elemental composition of the FEBID material derived by decomposition of the (H_3_Si)_2_Fe(CO)_4_ precursor was determined by EDX. The constant FEBID parameters included a deposition area of 1.4 μm × 1.4 μm, a 20 nm × 20 nm pitch, and a dwell time of 1 μs. The substrates for the EDX studies were sapphire single crystals coated with a 100 nm Au layer with a Cr (8 nm) adhesion layer. The FEBID material composition is plotted against (a) the acceleration voltage (1.6–2.4 nA; 1.0 × 10^–6^ mbar), (b) the beam current at a constant acceleration voltage of 5 kV and a total pressure of 1.0 × 10^–6^ mbar, and (c) the precursor feed represented by the total chamber pressure using 5 kV and 1.6 nA. (d–f) Plots showing the volume growth rates for different beam parameters and precursor feed. The inset in (e) shows cross-sectional shapes of FEBID deposits prepared at different beam currents. (g) AFM image of a typical 1.4 μm × 1.4 μm deposit (5 kV, 1.6 nA, 1.0 × 10^–6^ mbar) and (h) SEM image of a nanowire formed under spot deposition conditions (5 kV, 6.3 nA, 1.0 × 10^–6^ mbar). FEBID deposits prepared (i) at 5 kV and 6.3 nA on Pt microelectrodes located on micromembranes prepared at room temperature and (j) with temperature cycling of the micromembrane (0.1 s on, 0.5 s off), which illustrate similar deposits under both conditions.

Initial FEBID deposits of FeSi_*x*_-based materials were prepared under different beam conditions, varying the current and voltage of the electron beam. EDX analysis was carried out on 1.4 μm × 1.4 μm square deposits of at least 200 nm thickness that had been written onto Au-coated sapphire substrates. Figure 2a illustrates a decrease in metal/metalloid content from 44 to 38 at. % when the voltage is increased from 1 to 20 kV. The oxygen content remains at a constant level of approximately 30 at. %. However, the carbon content increases in this voltage regime from 25 to 32 at. %. This could be an effect of a lower number of near-surface electrons and a transition toward an electron-limited growth regime.

An inverse effect is observed for the variation in deposition current while keeping the beam voltage constant at 5 kV (Figure 2b). The metal/metalloid content increases from initially 39 at. % at 0.4 nA to 46 at. % at 6.3 nA, while the C content as well as the O content in the deposits decreases. It should be noted that the C:O ratio in the EDX measurements is typically close to 1, representing the negligible impact of background gases, such as water.^22^ The higher metalloid contents with increasing current are likely caused by an increased efficiency of carbonyl ligand liberation.

The highest metal/metalloid contents correspond to those parameters that show the highest growth rate per time (Figure 2d,e), potentially related to a shift toward the precursor-limited regime. Since the lower acceleration voltages provide higher numbers of secondary electrons close to the surface (Figure 2d), the regime is similar to the higher currents at a fixed acceleration voltage of 5 kV (Figure 2e). Interestingly, the precursor-composition-related Fe:Si ratio of 1:2 is retained reasonably well throughout the parameter range, even though a slight loss of silicon is observed, resulting in Fe:Si ratios ranging from 1:1.7 (0.58) to 1:1.5 (0.67). This is still very close to the CVD results using the same precursor and is illustrated in Figure 1b.

Overall intermediate growth rates ranging from 2 × 10^–3^ up to 6 × 10^–4^ μm^3^/nC are observed for voltages above 1 kV, similar to those reported for other binary M–Si-containing precursors.^39^ The decrease in growth rate observed for increasing currents correlates with a decrease in secondary electrons observed for a higher acceleration voltage (Figure 2d). With increasing electron energy, the penetration depth of electrons in the substrate material increases and less secondary electrons are able to reach the surface and contribute to the decomposition process.^61^ The evolution of growth rate observed for increasing current at a constant voltage of 5 kV is indicative of precursor-limited growth. The assignment of this deposition regime is also supported by the pressure-dependent growth rate in Figure 2f, which shows a slight increase of growth rate with pressure. Figure 2g,h shows different nanostructures grown by FEBID in this configuration, including the typical 2D patches as well as nanowires when single-spot deposition is used in the FEBID experiments.

The inset of Figure 2e shows cross-sectional AFM scans recorded for FeSi-based FEBID deposits written at 5 kV and a variation of the beam current, as indicated, while keeping the electron dose constant (half the dose for 0.4 nA). The regime of deposition for the deposits at 0.4 nA with a slightly higher deposit height at the edges indicates not only a diffusion-enhanced regime (DER) but also a precursor-limited one.^62,63^ Assigning a growth regime should include careful consideration of the potential influence of the growth strategy, here a serpentine patterning approach, when deposit morphologies are used for the interpretation and analysis.^64^ Very similar deposits have been observed using H_3_SiCo(CO)_4_.^39^ Higher currents show a consistent cross section with sharp edges.

In order to investigate whether temperature cycling can thermally complete the fragmentation process and liberate further CO ligands that had not been converted into reactive atomic species in the first steps of the electron-induced deposition process, additional FEBID experiments on micromembranes containing Joule heaters were carried out. For this purpose, the integrated microheater in the micromembrane substrates was alternatingly switched on for 0.1 s and off for 0.2, 0.5, 1.0, and 2.0 s during FEBID. Deposition at a constant elevated temperature is very slow due to accelerated precursor desorption; thus, cycling between on and off was required. The deposition dose was increased for the shorter off-cycles due to the overall longer heating times associated with lower FEBID growth rates due to the accelerated precursor desorption at elevated surface temperatures. The micromembrane current for Joule heating was chosen to be below the thermal decomposition temperature of the precursor. The deposition on the micromembranes with and without heating cycles was of similar resolution, as illustrated in Figure 2i,j. This indicates that buckling of the membrane is not an issue for the writing process during the thermocycling. However, the composition does not change when deposits with and without an additional heating step are compared, unless the membrane is heated above the thermal decomposition temperature, causing a simultaneous CVD deposition. The entering of the CVD window can be identified by recording the EDX spectra a couple of micrometers away from the FEBID material and comparing the Fe:Pt ratios. Even though these experiments did not show any changes in composition for the (H_3_Si)_2_Fe(CO)_4_ precursor investigated here, this platform can provide additional information concerning the formation of thermolabile intermediates and therefore is a very useful add-on to studying the properties of new precursors considered for FEBID applications.

Finally, the ion-induced direct-writing capabilities of this precursor were examined. In order to investigate FIBID using (H_3_Si)_2_Fe(CO)_4_, the deposition was carried out under variation of the ion beam current in the range between 1 and 30 pA and at voltages of 15–30 kV. Cu was used as the substrate surface, because the sputtering effects are much weaker when compared to those using a Au substrate material.^65^

**Figure 3:**
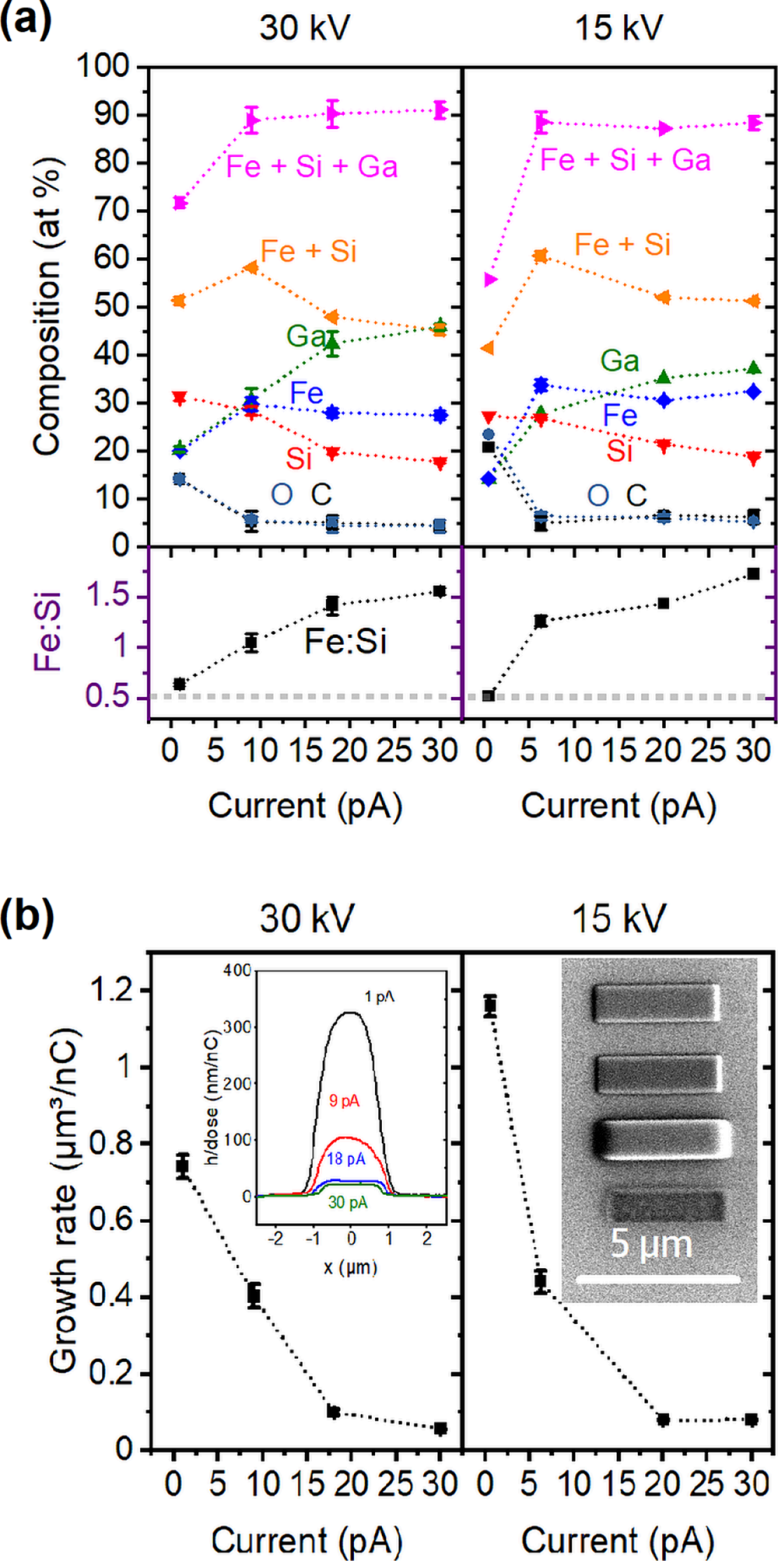
(a) Elemental composition of the FIBID material determined by EDX in relation to the beam current used (1–30 pA) at acceleration voltages of 30 and 15 kV. The lower part of the graph shows the Fe:Si ratio observed in the deposits. Further FIBID parameters include a deposition area of 1.4 μm × 1.4 μm, a 30 nm pitch in the *x* and *y* directions, and a dwell time of 0.2 μs. The substrates for the EDX studies are Cu-coated sapphire single crystals with a 200 nm layer thickness. (b) Growth rates of the FIBID material as calculated from the volume determined by AFM and the respective dose used. The growth rates are determined from nominal 1.0 μm × 5.0 μm patches grown at 30 and 15 kV using a 30 nm pitch in the *x* and *y* directions and a dwell time of 0.2 μs. Height profiles are shown in the inset of nominal 1.4 × 1.4 μm^2^ FIBID squares deposited at 30 kV in relation to the electron-beam current for an identical dose at higher currents (9–30 pA) and half the dose for 1 pA. The SEM image in the inset shows deposits written at 30 kV/10 pA, 15 kV/1 pA, 15 kV/3.5 pA, and 15 kV/0.5 pA (from top to bottom).

EDX analysis was carried out on 1.4 μm × 1.4 μm square deposits of at least 200 nm thickness on Cu-coated sapphire substrates. Figure 3a illustrates the compositional variation with increasing current and at constant acceleration voltages of 30 and 15 kV. The (H_3_Si)_2_Fe(CO)_4_ precursor leads to total metal/metalloid contents of 88–91 at. % in FIBID for currents above 5 pA. A lower metal/metalloid content of 56–72 at. % at the lowest current is accompanied also by a higher C and O content as well as a lower Ga content. These observations can be considered a consequence of sputtering effects of the low mass elements and a lower material growth efficiency with increasing currents. For the two acceleration voltages of 15 and 30 kV, the total percentage of Fe and Si peaks at 6.3–9 pA with 58–61 at. %. Therefore, deposits obtained with these settings were used for microstructural analyses as discussed below. The current-dependent FIBID studies were completed at a 5 kV acceleration voltage, even though the beam focus was rather broad. However, similar effects can be observed as for the higher voltages, but the sputtering and cleavage of CO leading to decreased C and O contents are less pronounced (Figure S2).

Momentum transfer from the incident Ga^+^ ions will contribute to the decomposition.^26^ Additionally, a much larger amount of secondary electrons is generated during ion impact when compared to FEBID, which could contribute to the decomposition of the precursor.^66^ The high overall metal/metalloid content of up to 91 at. % in FIBID is also a consequence of a very high Ga incorporation of ∼28 to 45 at. % in the deposits at beam currents of 9–30 pA.

A drastic increase in deposition efficiency per ion/electron of typically 2–3 orders of magnitude is reported for FIBID when compared to FEBID processes.^6,67^ The volume growth rate of the FeSi-based FIBID material is determined on SiO_2_ and shows a high deposition efficiency with values of 1.2–0.06 μm^3^/nC (Figure 3b). For (H_3_Si)_2_Fe(CO)_4_, about 2 orders of magnitude higher growth rate in FIBID for a similar precursor flux has been observed when compared to FEBID. The SEM image in the inset of Figure 3b shows typical FIBID deposits prepared at different growth conditions.

### Comparison of the Different Materials Obtained Depending on the Fragmentation Method

Overall, FIBID using (H_3_Si)_2_Fe(CO)_4_ leads to material with a C and O content well below 20 at. % approaching 5 at. % under optimized conditions while retaining a C:O ratio close to 1. However, an expedient comparison of CVD, FEBID, and FIBID can be based on the deposit composition per Fe atom deposited. Such a calculation reveals an Fe:C:O ratio for CVD of 1:0.1:0.1 (1 CO per 10 Fe); for FEBID, 1:1.41:1.45 (1 CO per 0.66 Fe); and for FIBID, 1:0.19:0.19 (1 CO per 5 Fe). In the FIBID deposits with lowest current (30 kV, 1 pA) and thus comparable Fe:Si ratio, the C and O contents are also reduced to 1:0.7:0.7 for Fe:C:O when compared to FEBID (5 kV, 6.3 nA) with 1:1.4:1.48. This effect could be related to a more effective CO abstraction in ion-induced deposition, while sputtering is still neglectable and similar to the previously reported surface science studies on metal carbonyls.^23,36^

Most notably, the Si content significantly decreases with increasing current for all FIBID acceleration voltages and currents higher than 1 pA. The reduced Si content can be attributed to the cosputtering effect competing with FIBID, where lighter atoms and atoms with a lower binding energy are sputtered more efficiently. Thermal or momentum-induced Fe–Si cleavage is rather unlikely since thermal CVD (Figure 1) shows no significant Si loss.

**Scheme 1:**
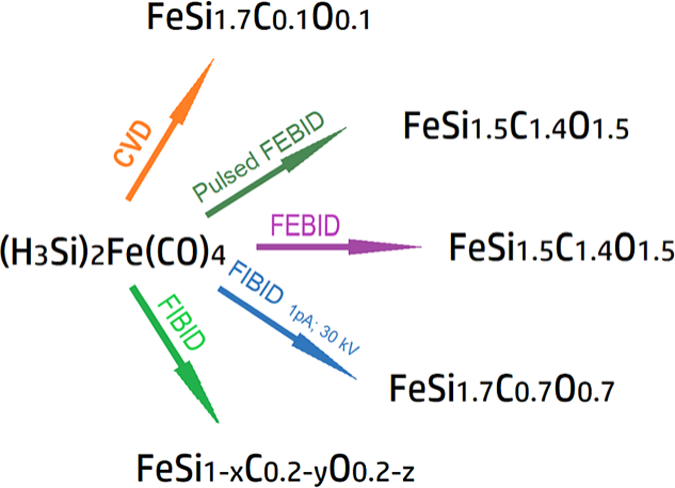
Summary of Compositions Achieved in the Different Gas-Phase Deposition Methods by Using the (H_3_Si)_2_Fe(CO)_4_ Single-Source Precursor Compositions correspond to CVD (573–773 K), thermal pulsing below the decomposition temperature, and simultaneous FEBID (5 kV, 6.3 nA), FEBID (5 kV, 6.3 nA), FIBID at lowest currents (30 kV, 1 pA), and generally FIBID at higher currents. Ga content in the FIBID material has been omitted.

Thermal decomposition via CVD retains 85% of the Fe:Si ratio in the deposit, while FEBID at 5 kV and 6.3 nA retains 79% and FIBID at 30 kV and 9 pA yields only 40% of the originally supplied Fe:Si ratio in the (H_3_Si)_2_Fe(CO)_4_ precursor. Consequently, sputtering should be considered the major contributing factor for Si loss in the FIBID results presented here since the lowest currents provide very similar Fe:Si ratios when compared to those of FEBID and CVD. Similar observations have been made for H_3_SiCo(CO)_4_, where significant Si loss has been reported. In contrast to these two precursors with terminally bonded SiH_3_ moieties, H_2_Si(Co(CO)_4_)_2_ owning a bridging silyl retains most of the Si and is less prone to sputtering effects, which could be related to either a higher growth rate or the bonding situation of the Si.^38^ This could indicate a general predicament of precursor design requiring multiple bonding of lighter elements in single-source precursors for binary materials in order to retain these elements in the ion-induced deposition. The compositions obtained by the different methods and processing conditions between the typical conditions applied are summarized in Scheme 1.

### Microstructural Characterization and Implications for (H_3_Si)_2_Fe(CO)_4_ Fragmentation

**Figure 4:**
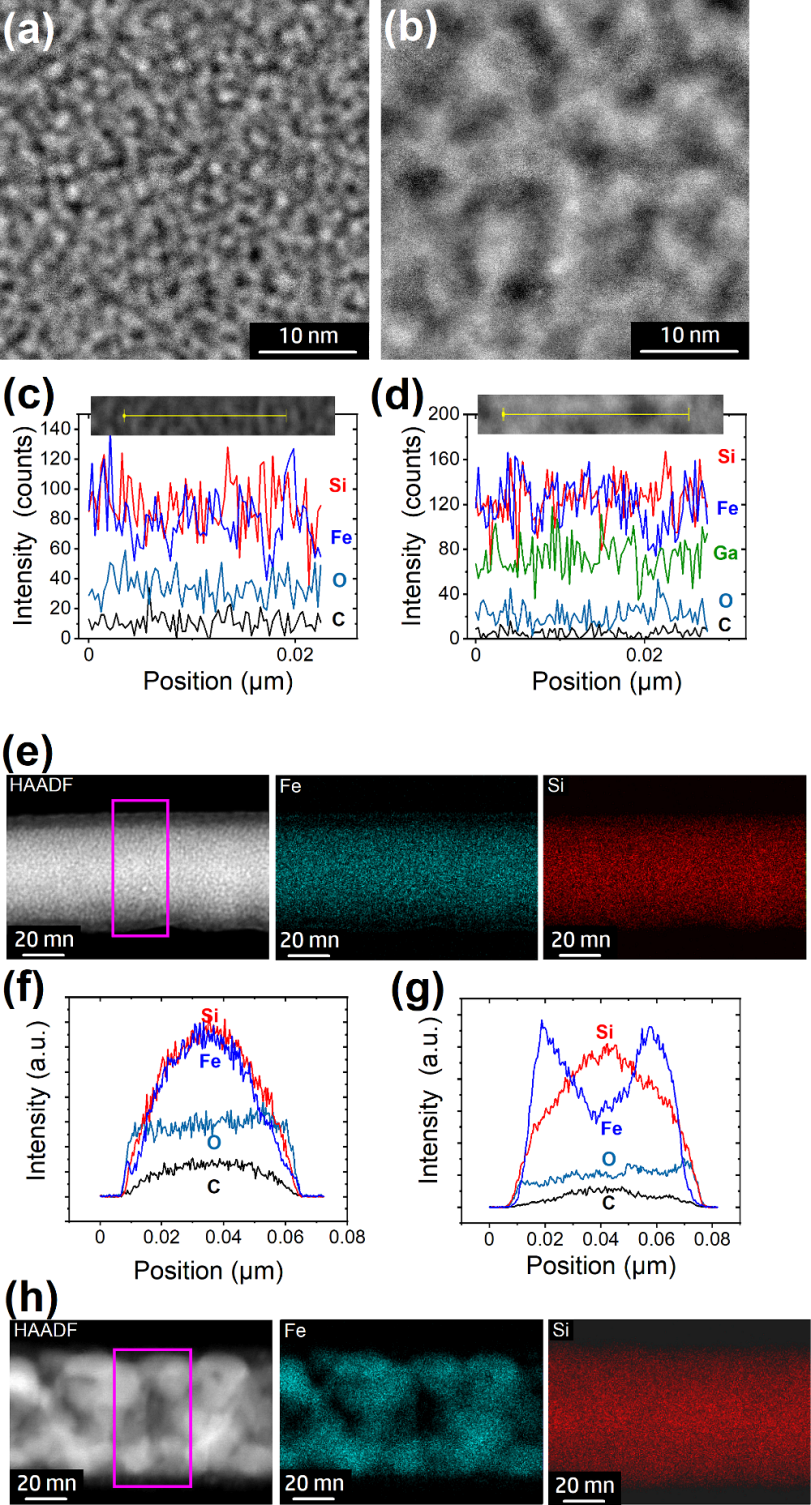
Representative HAADF images of (a) a FEBID (5 kV, 6.3 nA) and (b) a FIBID (15 kV, 3.5 pA) material prepared using the (H_3_Si)_2_Fe(CO)_4_ precursor on Cu-coated sapphire substrates. EDX line scans of the (c) FEBID and (d) FIBID materials with corresponding HAADF images illustrating the respective lines as insets. The effect of postgrowth electron-beam curing of the FEBID material is illustrated for a FEBID NW. (e) HAADF image and corresponding EDX Fe_K_ and Si_K_ maps and (f) areal cross-sectional EDX of the area marked by the pink box in (e) of the as-grown material. Cross-sectional elemental distribution after 10 min postgrowth electron-beam curing at 5 kV/6.3 nA: (g) areal cross-sectional EDX of the area marked by the pink box in (h) and (h) HAADF image and corresponding EDX Fe_K_ and Si_K_ maps.

TEM lamellae were prepared from (H_3_Si)_2_Fe(CO)_4_-derived FEBID and FIBID material. The HAADF TEM cross-sectional images of typical lamellae are shown in Figure 4a,b.

The FEBID material is rather homogeneous, with the brightness contrast revealing very small particles, which can be associated with nanoscale phase separation with a particle size below 2 nm. The line scan in Figure 4c reveals a rather homogeneous distribution of carbon and oxygen while small variations of Si and Fe are visible associated with the brightness contrast. The highly e-beam-sensitive nature of the FEBID material makes high-resolution elemental mapping in STEM mode very challenging for these lamellae since the material changes drastically during the investigation, as shown in Figure S3 of the Supporting Information.

Similarly, the FIBID material in Figure 4b,d reveals brightness contrast that can be associated with slight phase separation but much less sensitivity toward the electron beam during analysis. Darker areas in the HAADF image are slightly enriched in Si and O content, which can also be identified in the elemental maps for O and enrichment of Fe in the brighter sections (Figure S4 of the Supporting Information). This inertness to the electron beam of the FIBID material is most probably related to the high metallic content of ∼90 at. % and therefore a low tendency for SiO_*x*_C_*y*_ formation as a driving force for the phase separation.

In addition, FEBID nanowires (NWs) were used for further microstructural characterization. The homogeneous distribution of Si and Fe of the as-grown material is illustrated in the EDX maps as shown in Figure 4e and the areal line scan across the NW growth axis (Figure 4f). The Si_K_ and Fe_K_ signals correspond with a circular NW, which is also reflected in the C_K_ signal. Slightly higher oxygen signals at the edges are indicative of surface oxidation.

Figure 4g,h illustrates the effect of electron-beam curing (EBC) of a FEBID nanowire on the material’s microstructure. The as-grown FEBID material shows only feature sizes smaller than 2 nm, while the EBC-treated NW section reveals features in the ∼10 to 20 nm range. These changes are associated with Fe diffusion, while the Si_K_ signal distribution is very close to the one observed in the non-EBC-treated NW. Moreover, it should be mentioned that the Fe-dominated sections still contain a significant amount of Si, indicative of silicide formation.

Moreover, it should be mentioned that electron diffraction and fast Fourier transform (FFT) images from high-resolution (HR)-TEM images do not reveal any crystallinity in the FEBID and FIBID materials.

### Electrical Transport in FEBID and FIBID Materials

**Figure 5:**
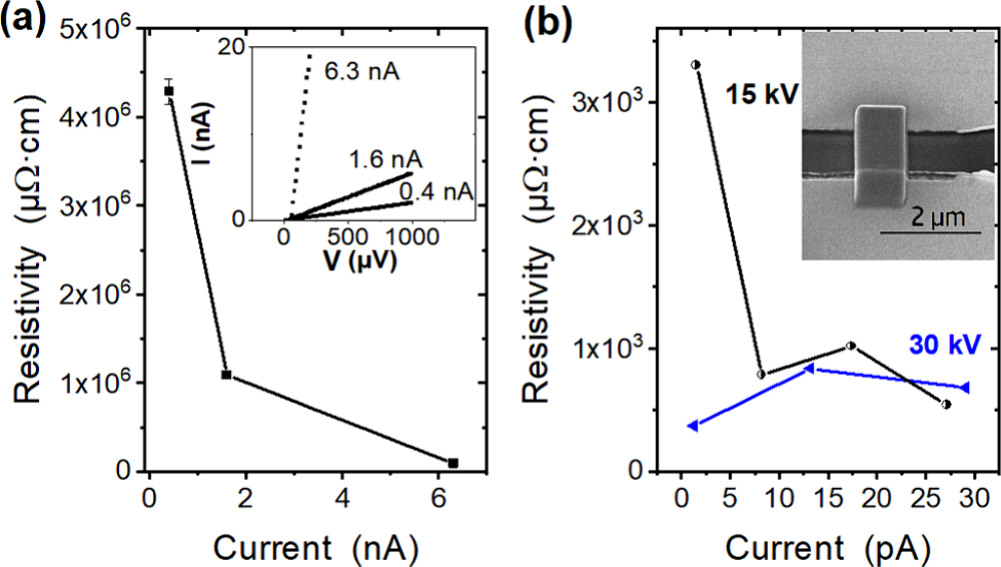
(a) Resistivity vs electron-beam current for two-probe FEBID material deposited at a 5 kV acceleration voltage and corresponding *I*/*V* curves as an inset. (b) Resistivity vs ion beam current that was used for the deposition of Fe–Si-based materials at acceleration voltages of 30 and 15 kV. The SEM image in the inset illustrates a typical deposit bridging two Au microelectrodes.

Electrical transport properties at room temperature were measured within the SEM chamber after the direct-writing process. *I*–*V* curves were recorded for the as-deposited material in two-point geometry. The FIBID and FEBID deposit bridged Au microelectrodes located on a SiO_2_ (300 nm) coated Si substrate, and the resistivities were deduced from the resistance values by using AFM results for the accurate determination of the deposit volumes. Figure 5 shows the resistivity values of the deposited material without corrections for potential contact resistances and contributions of the leads used, as these will have a negligible effect for the high-resistivity FEBID samples and lead to only small corrections for the low-resistivity FIBID samples. The FeSi-based material was deposited with various ion beam currents at constant acceleration voltages of 30 and 15 kV for FIBID as well as 5 kV for FEBID. The typical sample dimensions of FIBID structures for the two-terminal devices are 3 μm length, 1 μm width, and heights of 20–150 nm.

The FIBID material (∼3.2 × 10^3^ to ∼2 × 10^2^ μΩ·cm) generally shows resistivities lower than 1/30 of the best conducting FEBID material (∼4.3 × 10^6^ to ∼9 × 10^4^ μΩ·cm). Put simply, the tendency for reduced resistivity with increasing beam current could be associated with changes in composition (Figures 1 and 2). Moreover, the lower resistivities could be attributed to slight changes in microstructure with increasing ion/electron flux, similar to the often-observed effects in FEBID deposits,^68^ and/or a higher total metal content due to the increased Ga incorporation in FIBID.

It should be noted that the lowest resistivity values for other FIBID deposits derived by Ga^+^ ions based on Pt (∼800 μΩ·cm),^67^ Pd (∼1000 μΩ·cm),^69^ Co_2_Si (∼330 μΩ·cm),^38^ and W (∼200 μΩ·cm)^27,70^ typically are fairly high when compared to those of pure metals. Exceptions are the higher-purity Cu (∼50 μΩ·cm)^71^ and Co-based (∼20 μΩ·cm)^28^ FIBID material, but significant differences have been observed depending on compositional changes and postgrowth processing.

## Conclusions

In summary, decomposition processes of the (H_3_Si)_2_Fe(CO)_4_ precursor via CVD, FEBID, and FIBID differ significantly. While thermal decomposition retains the Fe:Si ratio well and results in more than 90 at. % metal/metalloid contents, the FEBID material contains between 40 and 50 at. % metal/metalloid and maintains the Fe:Si ratio. However, a strong microstructural variation is observed during further focused-electron-beam irradiation, leading to predominant Fe diffusion.

In contradistinction, FIBID material typically contains significantly lower Si contents for currents above 1 pA, which can be associated with sputtering and liberation of SiH_3_ moieties. An intermediate between the typical FEBID and FIBID materials is obtained at the lowest ion currents, which hints toward the possibility of deposition of high-purity metal silicide material retaining the Fe:Si ratio using other ion sources.

Importantly, the data reported on H_3_SiCo(CO)_4_, H_2_Si(Co(CO)_4_)_2_, and the here described (H_3_Si)_2_Fe(CO)_4_ precursors suggest well-retained metal:Si ratios in FEBID but, for FIBID, the loss of terminal SiH_3_ and retention of Si when bonded to more than one metal center.^38^ Therefore, precursor design should consider higher nuclearities as structural components in single-source precursors for FIBID.

Besides the in-plane deposition of nanostructures, 3D writing of nanowires using the (H_3_Si)_2_Fe(CO)_4_ precursor was demonstrated by FEBID. Finally, the applicability of micromembranes containing microheaters for the in situ study of thermal decomposition effects above room temperature is demonstrated and illustrates no significant loss of deposit resolutionwhen the micromembranes are thermally cycled during FEBID.

## Supporting Information Available

XRD of CVD deposits using the (H_3_Si)_2_Fe(CO)_4_ precursor and additional structural characterization such as TEM images and EDX maps (PDF)
