# Supplementary material for: Gas-Phase Synthesis of Iron Silicide Nanostructures Using a Single-Source Precursor: Comparing Direct-Write Processing and Thermal Conversion
Source: J Phys Chem C Nanomater Interfaces. 2024 Feb 8;128(7):2967–77. doi: 10.1021/acs.jpcc.3c08250 (PMC10910579; doi:10.1021/acs.jpcc.3c08250)
Supplement: Supplementary file 1 — jp3c08250_si_001.pdf [file jp3c08250_si_001.pdf]

Supporting Information

Gas Phase Synthesis of Iron Silicide Nanostructures using a Single-Source Precursor: Comparing Direct-Write Processing and Thermal Conversion

Felix Jungwirth[1,2], Alba Salvador-Porroche[3], Fabrizio Porrati[1], Nicolas P. Jochmann[1,2], Daniel Knez[4], Michael Huth[1], Isabel Gracia[5], Carles Cané[5], Pilar Cea[3,6], José María De Teresa[3], Sven Barth[1,2*]

[1] Institute of Physics, Goethe University Frankfurt, Max-von-Laue-Str. 1, 60323 Frankfurt am Main, Germany.

[2] Institute for Inorganic and Analytical Chemistry, Goethe University Frankfurt, Max-von-Laue-Str. 7, 60438 Frankfurt, Germany.

[3] Instituto de Nanociencia y Materiales de Aragón (INMA), CSIC-Universidad de Zaragoza, 50009 Zaragoza, Spain.

[4] Institute of Electron Microscopy and Nanoanalysis, Graz University of Technology, Steyrergasse 17, 8010 Graz, Austria.

[5] Institut de Microelectrònica de Barcelona (IMB), Centre Nacional de Microelectrònica (CNM), Consejo Superior de Investigaciones Científicas (CSIC), 08193 Barcelona, Spain.

[6] Laboratorio de Microscopías Avanzadas (LMA), Universidad de Zaragoza, Edificio de I+D+i, Campus Río Ebro, 50018 Zaragoza, Spain.

* corresponding author's email address: barth@physik.uni-frankfurt.de

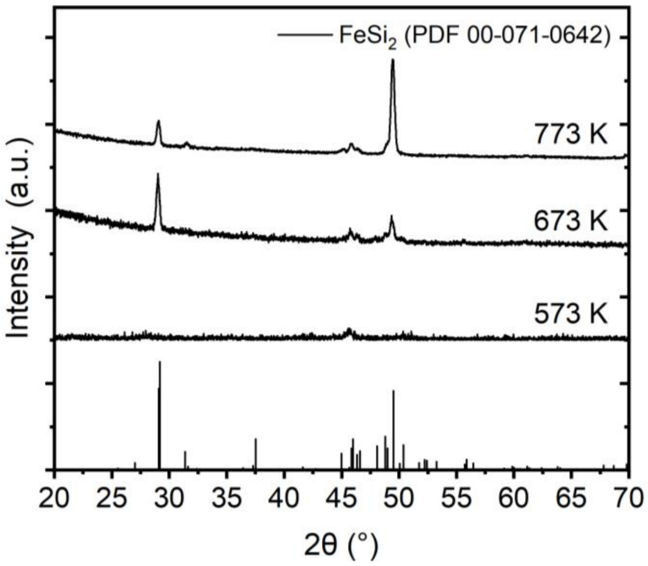

Figure S1: XRD pattern recorded at room temperature for films grown at substrate temperatures ranging from 573 to 773 K showing preferred orientation.

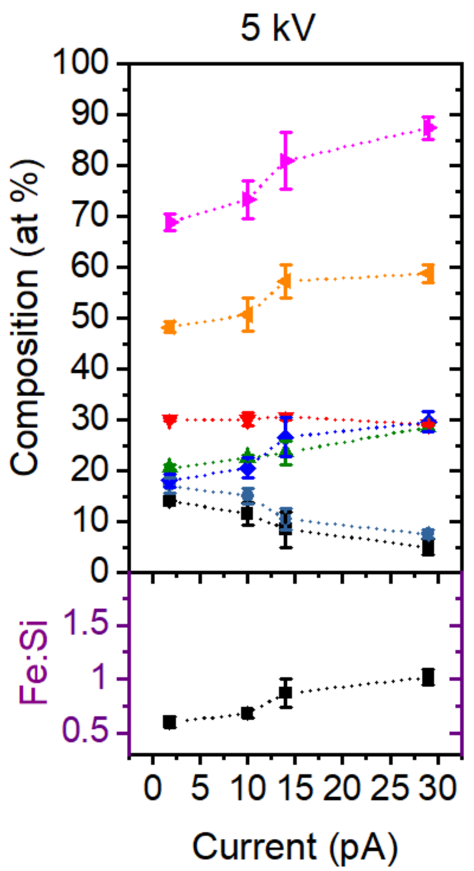

Figure S2: The elemental composition of the FIBID material determined by EDX in relation to the beam current used (1-30 pA) with an acceleration voltage of 5 kV. The lower part of the graphic shows the Fe:Si ratio observed in the deposits. Further FIBID parameters include the deposition area of 1.4 µm × 1.4 µm; a 30 nm pitch in x- and y-direction, and a dwell-time 0.2 µs. The deposition is carried out on Cu (200 nm) coated sapphire substrates.

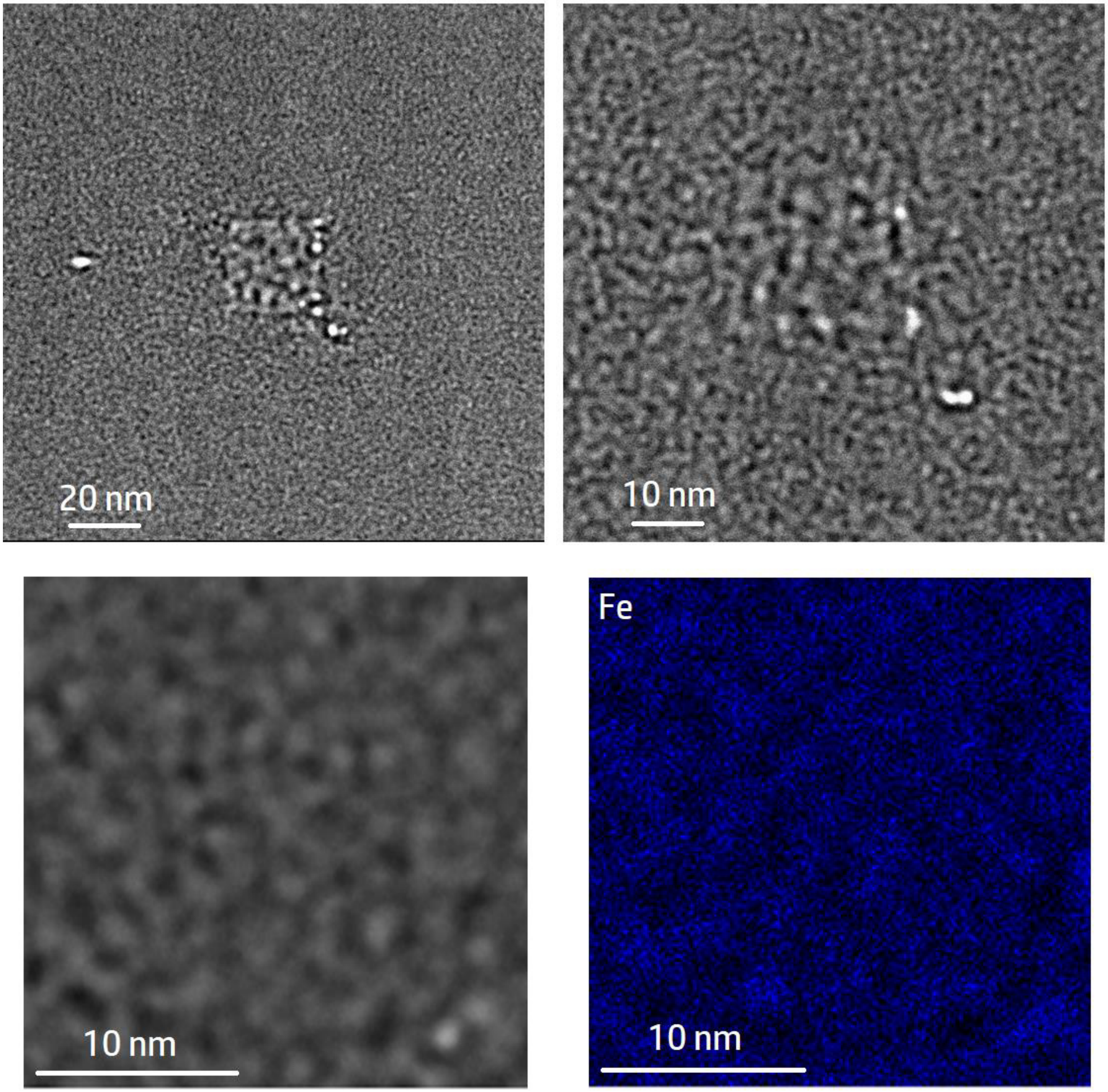

Figure S 3: HAADF images from FEBID lamellae showing the regions of the STEM mapping with altered microstructure due to electron beam exposure. Similarly, the HAADF taken before the mapping does not reflect the coarsened features observed in the Fe map.

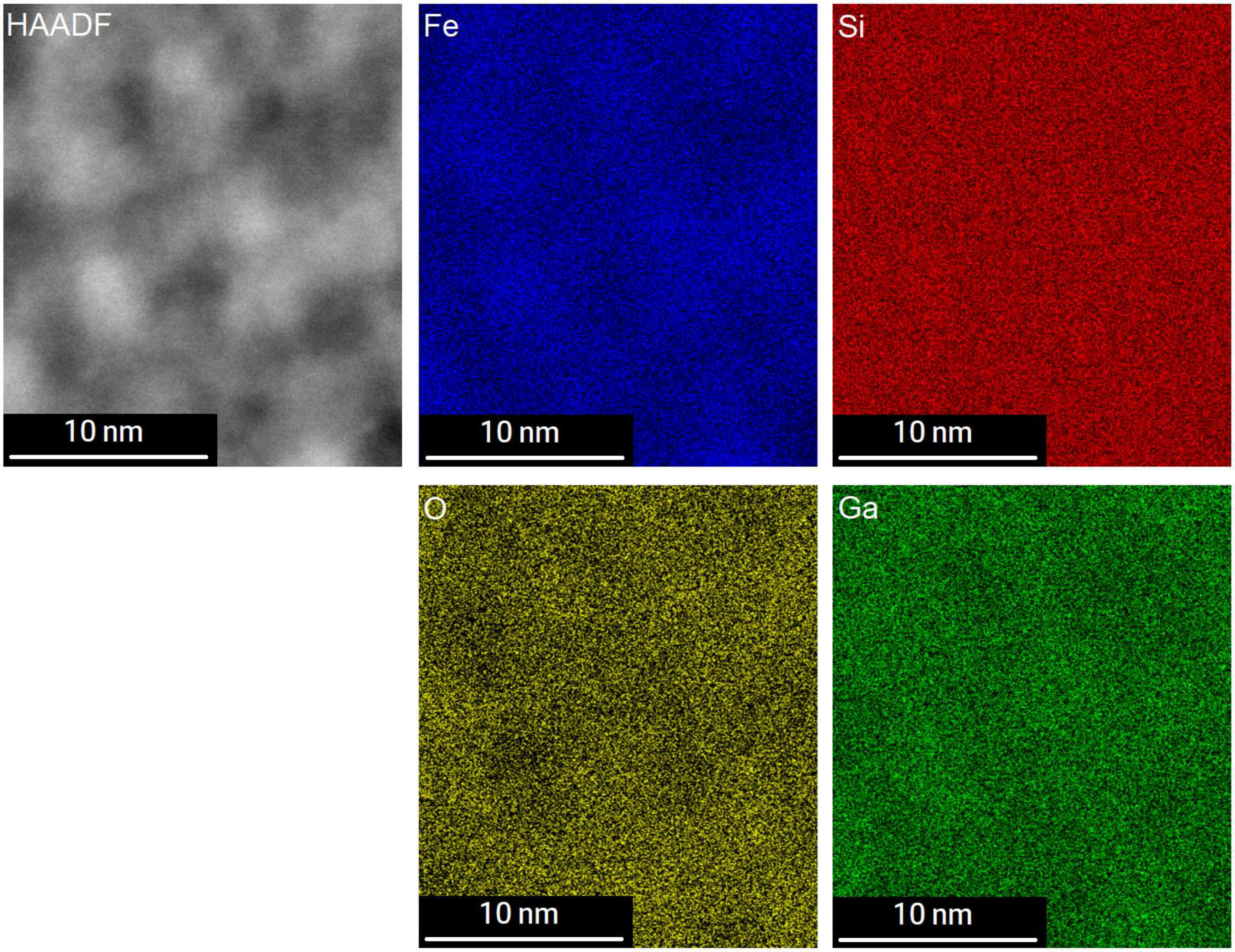

Figure S4: HAADF image and corresponding Fe_K, Si_K, O_K, and Ga_K map illustrating that the darker regions in the HAADF indeed reflect a lower concentration in Fe and slightly higher in oxygen.
